# Quality of Actions to Control Cervical Cancer in Bahia, Brazil

**DOI:** 10.31557/APJCP.2021.22.8.2343

**Published:** 2021-08

**Authors:** Eduarda Ferreira dos Anjos, Poliana Cardoso Martins, Nília Maria Brito de Lima Prado, Vanessa Moraes Bezerra, Patty Fidelis de Almeida, Adriano Maia dos Santos

**Affiliations:** 1 *Multidisciplinary Health Institute, Federal University of Bahia, Vitória da Conquista, Bahia, Brazil. *; 2 *Fluminense Federal University, Niterói, Rio de Janeiro, Brazil. *

**Keywords:** Primary health care, cervical cancer, papanicolaou test, screening, women’s health, quality of health care

## Abstract

**Objective:**

To assess the quality of the actions to control cervical cancer (CC) and its correlates.

**Methods:**

This is a cross-sectional study conducted from January to March 2019 in 19 municipalities in Bahia, Brazil, with a sample of 241 doctors and nurses from primary health care (PHC). Three dependent variables were chosen- “Performance of educational, promotion, prevention, and monitoring actions” (D1); “Access to diagnostic tests” (D2); “Non-occurrence of high grade cervical squamous intraepithelial lesions (HSIL)” (D3). Poisson regression with robust variance was used, adopting hierarchical input variables to estimate the prevalence ratios and confidence intervals of 95%.

**Results:**

The following prevalence rates were found: D1 39.8% (95% CI: 33.8-46.2); D2 73.9% (95% CI: 67.9-79.1); and D3 46.4% (95% CI: 39.9-53.0). These dimensions remained associated with the dependent variables: D1- having professional training courses on the topic; consideration to ensure that collection takes place appropriately by a professional; and women having access to medical transport; D2- nurses treating low-grade lesions; D3- recording the Papanicolaou in electronic medical records; D1 and D2- professionals joining the service through public tender; D1 and D3- working in the PHC (≥ 2 years); D2 and D3- recording Papanicolaou in physical records; and performance of Papanicolaou by residents.

**Conclusion:**

Better trained professionals and professionals working in stable work arrangements are associated with comprehensive actions to control CC. Such strategies indicate that investments in work management result in a more organized PHC and more solution-centered work processes. Therefore, working in the PHC for a longer time and nurses performing more clinical actions (collection and treatment) are favored by such organizational actions. Investments in diagnostic support contribute to perceptions of more comprehensive actions to control CC.

## Introduction

Cervical cancer (CC) is a global public health problem (GLOBOCAN, 2019), being the fourth with the highest incidence in the world. Low- and middle-income countries are the most affected, while in high-income countries, with early detection and treatment of high-grade intraepithelial lesions (HSIL), the incidence of CC is controlled (WHO, 2014; Arrossi et al., 2017). 

The World Health Organization (WHO) has established a goal to eliminate the disease (Canfell et al., 2020), through the expansion of vaccinations against human papilloma virus (HPV), cervical screening, and treatment of cases of HSIL and CC (Arrossi et al., 2017), associated with programs to ensure the quality of these actions. For this, the existence of a multidisciplinary team (WHO, 2014) with stable working ties (Muramoto and Matumoto, 2019), attentive work of community health agents- CHA (Taylor et al., 2010; Fernandes et al., 2019), care coordination (Vázquez et al., 2017), integration between teaching and service (Baldoino and Veras, 2016), as well as adaptation of the structure (Bottari et al., 2008) and of the work process (Barcelos et al., 2017) are associated with quality in health actions, thus becoming central to the control of long-term illnesses such as CC.

Within the scope of programs for the control of CC, for complex scenarios such as the Brazilian one, healthcare networks are essential to offer care in a timely and comprehensive manner, as they allow longitudinal care, which is appropriate for Brazil and has greater resolution (Brito-Silva et al., 2014). Thus, the line of care of CC makes it possible for the user to walk through the services that must be provided at the different levels of the network, by means of continuous streams that provide full care (Galvão et al., 2019).

Thus, this article assesses the quality of actions to control cervical cancer and its correlates, in the interior of Bahia, Brazil.

## Materials and Methods

*Methods*

Cross-sectional research conducted in 19 municipalities (641,560 inhabitants) in the interior of Bahia, Brazil, from January to March 2019. CC was used as a tracer condition to assess actions necessary for the quality of care (Bottari et al., 2008) provided by PHC professionals. These municipalities cover a large part of the population living in rural areas and, therefore, many professionals working in large rural areas with great social inequality.

*Population studied*

The sample consisted of 354 doctors and nurses. A prevalence of 50% for unknown events and a confidence level of 95% were used, obtaining a minimum sample of 240 professionals, considering 30% for losses. A random draw was performed considering the number of professionals registered in the PHC teams in each municipality. Professionals who were not working (vacation, leave, etc.) during the study period were excluded.

Individual interviews were conducted using questionnaires. The questionnaire was adapted from an instrument used to assess care coordination in health regions (Souza et al., 2015), to which information from national protocols were included (Brazil, 2013; Brazil, 2016).

*The quality of actions*

Three dependent variables were chosen to evaluate the actions developed to control CC in the day-to-day of professionals: “Performance of educational, promotion, prevention, and monitoring actions” (D1); “Access to diagnostic tests” (D2); “Non-occurrence of HSIL” (D3). These variables represent some of the criteria for the actions required by the health service system to ensure a quality program for CC control.

D1- obtained from the combination of educational and monitoring actions: “Conducting home visits to mobilize women to carry out preventive measures”; “Carrying out health education actions”; “Conducting joint efforts to expand access to cytopathological examination”; “Recording women who underwent examination at the PHC”; “monitoring the record to identify women with delayed or altered exams” and “Active search of women with delayed collection or altered results.”

The following promotion and prevention actions were also considered: “Cytopathological examination collection” and “Women’s access to colposcopy.” The response options for the actions were “yes/no.” Positive responses were used for the event, and all actions were classified as being of adequate quality, since they are constituent elements of a comprehensive CC prevention and control program (Brazil, 2013; WHO, 2014; Brazil, 2016).

D2- “Access to diagnostic tests”- obtained from the combination of responses to actions perceived by professionals regarding “Access to women with lesions prior to biopsy” and/or “Access of women with lesions prior to histopathology.” The responses were dichotomized into “always/sometimes or never.” The answer “always” was used in all questions, as access to diagnostic tests are fundamental to stop the development of CC and, therefore, to achieve the desired quality (Brazil, 2013; Brito-Silva et al., 2014; WHO, 2014; Brazil, 2016).

D3- “Non-occurrence of HSIL”- It was considered that a quality program can prevent the occurrence of high-grade lesions (Brazil, 2013; Goss et al., 2013; WHO, 2014; Brazil, 2016). This dependent variable was obtained by questioning about the “Presence of women diagnosed with HSIL in the PHC,” whose response options were “yes/no,” using negative answers.

*Independent variables*

**Figure 1:**
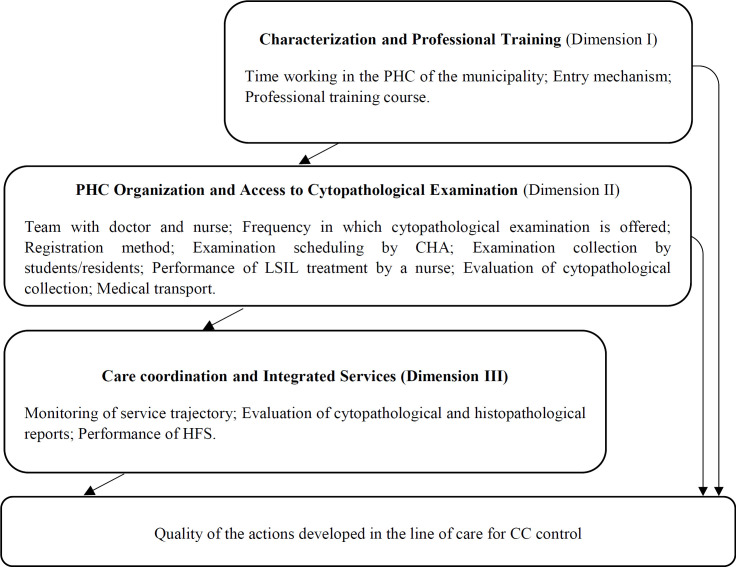
Hierarchical Conceptual Model of Quality Analysis of the Actions Developed in the Line of Care for Cervical Cancer Control. Abbreviations: PHC, Primary Health Care; CHA, Community Health Agents; LSIL, Low Grade Squamous Intraepithelial Lesion; HFS, High Frequency Surgery; CC, Cervical Cancer

The selected independent variables are shown in Figure 1, according to the blocks “Characterization and professional training,” “Organization of the unit and access to cytopathological examination,” and “Care coordination and integrated care.”

*Statistical analysis*

Descriptive analyses were obtained using absolute and relative frequency measurements. For bivariate analysis, the differences between the proportions were assessed by the χ2 Pearson test. The analysis of factors correlated to the dependent variables (D1, D2 and D3) was performed using Poisson regression with robust variance, estimating the prevalence ratio (PR), p-value, and the 95% confidence interval (95% CI). Variables with a significance level ≤0.20 were included in the multivariate model.

Possible correlations between independent variables and D1, D2 and D3 were measured using different models adjusted through the hierarchical entry of the variables (Figure 1). Initially, the association between each dependent variable and the dimension I variables were evaluated, the dimension II variables were included in the second model, and the dimension III variables were included in the third model. The models were compared by the Akaike’s Information Criterion (AIC) test. 

The variables of the most distal blocks remained as adjustment factors for the hierarchically lower blocks. For the interpretation of the results, the identification of a statistically significant association (p ≤ 0.05) between each factor studied and the D1, D2 and D3, after adjusting for the potential factors of the same block and the upper hierarchical blocks, indicate the existence of an independent effect, specific to that factor. The Stata statistical package version 15.0 was used for data analysis.

The research was approved by the Research Ethics Committee of the Federal University of Bahia (opinion No. 624,168).

## Results

The prevalence of D1 was 39.8% (95% CI 33.8-46.2), whereas for D2 and D3, it was 73.9% (95% CI 67.9-79.1) and 46.4% (95% CI 39.9-53.0), respectively. 

About 109 doctors and 132 nurses were interviewed. Table 1 describes the PHC teams. Regarding the length of experience, 56.4% of professionals were in PHC for ≥ 2 years and 43.6% reported having a inadequate employment model, through political indication or other precarious methods of entry. Regarding training, 59.6% reported having participated in professional courses addressing the topic (Table 1).

With regard to the way the PHC is organized to provide access to users, almost all of them had doctors and nurses (94.6%), the highest frequency in which cytopathological examination was offered was at least weekly (67.7%) and the main method of recording was the simultaneous use of physical and electronic medical records (40.7%). Most CHA scheduled cytopathological examinations (90.4%), students/residents did not perform the collection even when accompanied by their preceptors (83.1%), and nurses did not treat low-grade lesions (62.1%). The evaluation of examination collection was positive (77.7%) and medical transportation was accessible to women (65.5%) (Table 1).

The professionals monitored the service trajectory of women, verifying adherence to treatment at the specialized service (76.4%), the cytopathological and histopathological reports issued by the reference laboratory were reliable (59.4%), and women had access to high-frequency surgery (HFS) (71.0%) in cases of lesions being confirmed after colposcopy (Table 1).

In the bivariate analysis, in the dimension of “Characterization and professional training,” there was a positive association between: time working in PHC ≥ 2 years (D1 and D3); having entered through public tender (D1 and D2), and having professional training on the topic (D1) (Table 1).

In the dimension “PHC organization and access to cytopathology,” the correlations were: form of registration (D3), collection by students (D1, D2 and D3), and treatment of low grade squamous intraepithelial lesion (LSIL) by a nurse (D1 and D2). The professional evaluation of the collection as adequate (D1 and D2) and the woman having access to medical transportation (D1) (Table 1).

In the dimension “Care coordination and integrated services,” the associations were: professionals who followed the service trajectory of the women verifying adherence to treatment in the specialized service (D1 and D2), evaluation of the reports issued by the reference laboratory as reliable (D1) and women having access to HFS (D1) (Table 1). 

In the multivariate analysis (Table 2), the following remained correlated: working in PHCs in the municipality (≥ 2 years) (D1 and D3), having joined the service through public tender (D1 and D2), having professional training on the topic (D1), the recording of cytopathological examinations using a physical medical record (D2 and D3) or an electronic medical record (D3), collection of the cytopathological examination by students (D2 and D3), nurses performing LSIL treatment (D2), evaluation of the cytopathological collection as adequate (D1), and women having access to medical transportation (D1).

**Table 1:** Sample Description and Bivariate Analyzes between D1, D2, D3 and Dimension I, II, and III Variables, Bahia, Northeast, Brazil, 2019

Variable	Sample distribution	CC educational, promotion, prevention, and monitoring actions		Access to diagnostic tests		Non-occurrence of HSIL	
	N (%)	P (%)	p-value	P (%)	p-value	P (%)	p-value
Characterization and professional training (dimension I)							
Occupation			<0.001		0.303		0.146
Physician	109 (45.2)	23.9		77.1		52.2	
Nurse	132 (54.8)	53.0		71.2		42.3	
Time working in the PHC of the city			<0.001		0.053		0.001
< 2 years	105 (43.6)	22.9		67.6		59.3	
≥ 2 years	136 (56.4)	52.9		78.7		37.4	
Entry mechanism			<0.001		0.051		0.048
Indication or other	105 (43.6)	32.4		69.5		50.0	
Public selection	83 (34.4)	31.3		71.1		52.1	
Public tender	53 (22)	67.9		86.8		31.4	
Professional training course			<0.001		0.074		0.059
No	97 (40.4)	25.8		68.0		54.0	
Yes	143 (59.6)	49.7		78.3		41.0	
PHC organization and access to cytopathological examination (dimension II)							
Team with doctor and nurse			0.144		0.696		0.269
No	13 (5.4)	61.5		69.2		30.8	
Yes	228 (94.6)	38.6		74.1		47.4	
Frequency in which cytopathological examination is offered			0.316		0.637		0.991
Monthly	22 (9.9)	27.3		72.7		44.4	
Fortnightly	50 (22.4)	42.0		80.0		44.9	
Weekly or more frequently	151 (67.7)	44.4		73.5		45.7	
Registration method			0.367		0.088		0.024
Both	98 (40.7)	34.7		66.3		57.1	
Physical medical record (on paper)	48 (19.9)	42.1		79.2		35.6	
Electronic medical record	95 (39.4)	45.8		79.0		40.7	
Examination scheduling by CHA			0.162		0.493		0.686
No	22 (9.6)	27.3		81.8		50.0	
Yes	206 (90.4)	42.7		75.2		45.5	
Examination collection by students/residents			<0.001		0.004		0.005
No	197 (83.1)	35.0		71.1		50.6	
Yes	40 (16.9)	65.0		92.5		25.6	
LSIL treatment performed by a nurse			0.001		0.003		0.058
No	146 (62.1)	32.2		67.8		50.8	
Yes	89 (37.9)	53.9		85.4		37.7	
Assessment of cytopathological examination collection			0.001		0.053		0.973
Inadequate or partially adequate	52 (22.3)	21.2		65.4		45.8	
Totally adequate	181 (77.7)	46.4		78.5		45.6	
Medical transport			0.001		0.143		0.682
Never or sometimes	69 9 (34.5)	26.1		66.7		47.5	
Always	131 (65.5)	50.4		76.3		44.4	
Coordination of care and integrated services (dimension III)							
monitoring of service trajectory			0.016		0.047		0.058
Never or sometimes	55 (23.6)	27.3		65.5		57.1	
Always	178 (76.4)	45.5		78.7		41.8	
Evaluation of cytopathological and histopathological reports			0.013		0.215		0.765
Partially reliable or I do not trust	95 (40.6)	30.5		70.5		46.6	
Totally reliable	139 (59.4)	46.8		77.7		44.5	
Performance of HFS			<0.001		0.140		0.313
Never or sometimes	45 (29.0)	20.0		75.6		47.5	
Always	110 (71.0)	56.4		85.5		38.3	

CC, Cervical Cancer; HSIL, High-Grade Intraepithelial Lesion; PHC, Primary Health Care; CHA, Community Health Agent; LSIL, Low-Grade Squamous Intraepithelial Lesion; HFS, High-Frequency Surgery

**Table 2:** Multivariate Analysis of Factors Correlated with “CC Educational, Promotion, Prevention, and Monitoring Actions,” “Access to Diagnostic Tests,” and “Non-Occurrence of HSIL,” Bahia, Northeast, Brazil, 2019

Variable	CC educational, promotion, prevention, and monitoring actions		Access to diagnostic tests		Non-occurrence of HSIL	
	PR	95% CI	PR	95% CI	PR	95% CI
Characterization and professional training (dimension I) *						
Time working in the PHC of the city						
< 2 years	1.00				1.00	
≥ 2 years	1.75	1.15-2.66			1.54	1.16-2.04
Entry mechanism						
Indication or Other	1.00		1.00			
Public selection	0.93	0.62-1.40	1.02	0.85-1.23		
Public tender	1.52	1.07-2.15	1.25	1.06-1.47		
Professional training course						
No	1.00					
Yes	1.49	1.02-2.18				
PHC organization and access to cytopathological examination (dimension II) †						
Registration method						
Both			1.00		1.00	
Electronic medical record			0.91	0.74-1.11	1.43	1.05-1.95
Physical medical record (on paper)			0.77	0.63-0.94	1.60	1.15-2.22
Examination collection by students/residents						
No			1.00		1.00	
Yes			1.32	1.11-1.56	1.65	1.21-2.25
LSIL treatment performed by a nurse						
No			1.00			
Yes			1.18	1.02-1.37		
Assessment of cytopathological examination collection						
Inadequate or partially adequate	1.00					
Totally adequate	2.00	1.06-3.75				
Medical transport						
Never or sometimes	1.00					
Always	1.61	1.04-2.48				
Care coordination and integrated services (dimension III) ‡						
Akaike criteria	3.558.913		4.683.768		3.854.261	
	2.964.822		4,578,194		3.803.587	
	2.964.822		4,578,194		3.803.587	

CC, Cervical Cancer; HSIL, High-Grade Intraepithelial Lesion; PHC, Primary Health Care; LSIL, Low Grade Squamous Intraepithelial Lesion. * Adjusted between the dimension I variables; † Adjusted between the dimensions I and II variables; ‡ Adjusted between the dimensions I, II, and III variables.

## Discussion

Our findings suggest a moderate quality in actions necessary for comprehensiveness for CC control, since the diagnostic access, although surprisingly high, coexisted with the frequent occurrence of LSIL. Contradictorily, prevention and monitoring actions had a low prevalence. Therefore, there are indications that it is necessary to improve the educational and monitoring actions of CC, which, in turn, could led to a decrease in cases of HSIL and, to a large extent, of CC (Romli et al, 2020). Furthermore, the screening program and the early detection of lesions (diagnosis) may have no impact on the incidence of CC if they are not associated with treatment (WHO, 2014; Sarfati et al., 2019).

The high turnover of doctors is a major problem in Northeast Brazil (Gonçalves et al., 2019) resulting from the greater concentration of resources and services in capitals and regional hubs (Albuquerque et al., 2017), which compromises timely access and continuity of care.

Working for a longer time in PHCs in the same municipality and having joined the service through public tender represent a lower professional turnover and better labor links, allowing the formation of a community link (Muramoto and Matumoto, 2019). Furthermore, the establishment of trust with professionals increases the adherence of women and the resolution of cases, especially for care associated with sexuality, which, due to moral values, can interfere with the perception of risk and become a symbolic barrier to access (Rico and Iriart, 2013). Furthermore, satisfaction with work and wages favor care coordination (Vázquez et al., 2017).

The training of professionals through the provision of in-service education is a recommendation of global and national guidelines for better quality and resoluteness in services provided to the community (WHO, 2014; Brazil, 2016). Well-trained professionals guide their actions by safe evidence and gain confidence in clinical practices, mitigating unnecessary referrals and improving PHC attributes (Leão and Caldeira, 2011). 

Regarding PHC organization and access to cytopathological examination, the registration method is an aspect of the work dynamics that allows for more adequate tracking, in addition to better management and informational coordination of care. The use of electronic medical records represents an advance in the communication mechanisms resulting in greater satisfaction and trust of users (Wali et al., 2020). Better communication between care levels is crucial for chronic conditions in systems with limited resources (Sarfati et al., 2019), as they facilitate care (Rahal et al., 2019), allowing longitudinal and articulated monitoring.

The performance of the examination collection by students/residents is a strong indicator of the integration between teaching and service, which promotes a better quality of service, because, in addition to providing greater access to screening exams, it also allows the service professionals to be up to date. Furthermore, it encourages the early development of professional skills and proactive attitudes of students, enables the training of professionals committed to innovation and adequate performance in complex contexts, combining theory and practice, which are essential for critical thinking and a broader view of care in health (Botti and Rego, 2011; Baldoino and Veras, 2016).

The performance of LSIL treatment by a nurse shows the relevance of this professional for the operationalization of cases in PHC (Fernandes et al., 2019). This is encouraged in different countries to expand the scope of actions in PHC and the ability to meet users’ needs (Toso et al., 2016). The involvement of nurses in screening increases the confidence and adherence of vulnerable women (Perks et al., 2018).

The professional assessment of the collection of the cytopathological examination is an indicator that can provide data on the quality of teamwork and the confidence of professionals in their co-workers. Services where there is greater connection and a better working environment among the team tend to be more innovative, resolute, and better evaluated by users, especially with chronic conditions (Proudfoot et al., 2007). A better work environment results in integration and motivation for work, as well as reciprocity between team members and forging a collaborative network (Peruzzo et al., 2019).

The access of women to medical transport is fundamental for the quality of services in a network with a strong governance and logistical system, since the treatment of CC, and in some cases of HSIL, is carried out outside the municipality of residence. CC mainly affects women with lower incomes and from rural areas (Goss et al., 2013), thus, medical transport is an organizational component for reducing access inequities, decreasing user absenteeism.

For this article, analyzes were performed with hierarchical entry of the variables, which is a strategy to deal with a greater number of explanatory variables. The inclusion of the variables and the conceptual model constructed considered the data available in the national (Brazil, 2016) and international literature (WHO, 2014). It is highly likely that these data can be generalized, considering most small municipalities in Brazil, even to networks with similar contexts, especially in Northern and Northeastern Brazil. Studies in small municipalities in Northeastern Brazil are crucial for increasing the understanding and promoting interventions in the public health system, which will make it more comprehensive and make CC control feasible.

## Data Availability

All data generated or analysed during this study are in the custody of the researchers and will be made available upon request.

## Origin of the article

This article was extracted from the thesis entitled “Evaluation of the Quality of the Cervical Cancer Control Actions in a Health Region of Northeastern Brazil”.
